# Macronutrient Sensing in the Oral Cavity and Gastrointestinal Tract: Alimentary Tastes

**DOI:** 10.3390/nu13020667

**Published:** 2021-02-19

**Authors:** Russell Keast, Andrew Costanzo, Isabella Hartley

**Affiliations:** CASS Food Research Centre, School of Exercise and Nutrition Sciences, Deakin University, Burwood, VIC 3125, Australia; andrew.costanzo@deakin.edu.au (A.C.); b.hartley@deakin.edu.au (I.H.)

**Keywords:** taste, obesity, fat, protein, carbohydrate

## Abstract

There are numerous and diverse factors enabling the overconsumption of foods, with the sense of taste being one of these factors. There are four well established basic tastes: sweet, sour, salty, and bitter; all with perceptual independence, salience, and hedonic responses to encourage or discourage consumption. More recently, additional tastes have been added to the basic taste list including umami and fat, but they lack the perceptual independence and salience of the basics. There is also emerging evidence of taste responses to kokumi and carbohydrate. One interesting aspect is the link with the new and emerging tastes to macronutrients, with each macronutrient having two distinct perceptual qualities that, perhaps in combination, provide a holistic perception for each macronutrient: fat has fat taste and mouthfeel; protein has umami and kokumi; carbohydrate has sweet and carbohydrate tastes. These new tastes can be sensed in the oral cavity, but they have more influence post- than pre-ingestion. Umami, fat, kokumi, and carbohydrate tastes have been suggested as an independent category named alimentary. This narrative review will present and discuss evidence for macronutrient sensing throughout the alimentary canal and evidence of how each of the alimentary tastes may influence the consumption of foods.

## 1. Introduction

The alimentary canal comprises various organs including the mouth, throat, esophagus, stomach, intestines, and anus and encompasses a system that is responsible for identifying foods suitable for consumption, preparation for swallowing, digestion, absorption, and finally excretion of waste. Put simply, the alimentary canal can be considered a mouth to anus nutrient (and non-nutrient) recognition and processing system. It appears logical that we have sensing systems that respond to the macronutrients fat, protein, and carbohydrate. For each macronutrient, there are two perceptual outcomes (at least), one for the monomer, one for larger compounds: fat has a fat taste for the monomer fatty acid (FA), and mouthfeel for triacylglycerol (TAG) compounds; protein has umami for the monomer L-glutamate and kokumi for γ -glutamyl peptide compounds; carbohydrate has sweet for the monomer sugar, and carbohydrate for oligosaccharide compounds.

Within the alimentary canal, there are two distinct areas of signaling nutrient composition, the upper alimentary canal, comprising the mouth and pharynx, where recognition of various food chemicals signals basic taste qualities (sweet, sour, salty, and bitter), and the lower alimentary canal comprising the stomach and small intestine where digestion of protein, carbohydrate and fat is completed and the absorption of nutrient occurs. What follows is a brief overview of the taste system, and a narrative review of the non-traditional tastes responding to macronutrients, from pre-ingestion to post-ingestion.

## 2. Taste

Sensing of foods by the taste system has been critical for species survival, signaling via taste quality (and hedonic response) whether the food is fit for ingestion. As an example, if potential food is excessively bitter or sour, ingestion is discouraged via a negative hedonic response and the food would be rejected. The basic taste system is a robust initial screening protecting the digestive system from foods that may be harmful whereas sweet and salty signals both encourage ingestion via a positive hedonic response. The ability to swiftly assess the suitability of foods for consumption has been vital during the successful evolution of species.

### 2.1. Basic Taste

Taste research is ever evolving through advancements in psychophysical research and molecular biology, particularly the discovery of taste receptors. Traditionally and throughout history, there have been four tastes considered to be the basics: sweet, sour, salty, and bitter. Due to their perceptual salience, these four tastes have been consistently named in taste lists across several thousands of years and multiple cultures (Greek, Aristotle; ancient Chinese medicine, and Indian medicine, Ayurveda) [1,2]. These basic tastes have an important influence on the nutritional or toxic status of the food when it enters the oral cavity [3,4,5], and have unmistakable perceptual salience. It is this clear perceptual salience and historical categorization of these four independent tastes that has stood the test of time and solidifies these tastes as the basic tastes [1,2]. 

### 2.2. Measuring Taste

In human studies, measures of taste function include detection threshold (DT), the lowest concentration of a stimulus that is perceivable, recognition threshold (RT), the concentration at which the quality of the stimuli can be correctly identified, and the suprathreshold intensity range, which increases with increasing stimuli concentration to a terminal threshold [6]. These three measures are all reflective of taste’s perceptual domain, but studies have illustrated that the measures are not necessarily correlated with each other. For example, an individual who has a low detection threshold for sucrose (sweet) is termed hypersensitive, but the same individual may experience low intensity of sweetness at a higher concentration of sucrose and be termed hyposensitive [7]. Therefore, within a basic taste quality, an individual may be classified as more or less sensitive. To further confound, there may be a lack of association in taste sensitivity between compounds that elicit the same quality, for example, an individual may have different taste sensitivity to sucrose and acesulfame K which are both sweet stimuli. The same principle applies across taste qualities, and just because an individual is termed hypersensitive to a bitter compound (e.g., 6-n-propylthiouracil) does not predicate that they will be sensitive to sucrose [8]. That the basic tastes are not correlated may be a reason for the lack of consistency between studies when assessing basic taste sensitivity and dietary consumption [9]. However, the development of taste research beyond the four basic tastes provides many avenues for future research.

## 3. Beyond Basic Tastes: Alimentary Tastes

Advancements in molecular biology and the discovery of taste receptors that detect specific taste stimuli have begun to broaden the initial four independent tastes to potentially include a myriad of new tastes, which do not have the same perceptual salience as the basic tastes. For example, in the early 2000s, umami taste, and more recently, fat taste, have been added to this basic taste list based on the discovery of receptors specific to umami [10] and fat stimuli [11]. However, due to more recent advancements in technology, the future discovery of taste receptors that respond to other taste stimuli such as γ-glutamyl peptides (kokumi) [12] and oligosaccharides (carbohydrates) is inevitable. Thus, the four basic tastes (sweet, sour, salty, and bitter) that have been solidified across several thousands of years are in a class of their own, and new tastes predominately discovered by molecular and modern psychophysical research should be considered in a new subgroup of tastes, for example, alimentary tastes, which emerging evidence suggests have an influence on diet via post-ingestive consequences [2]. Of main relevance to this topic, the gastrointestinal tract (GIT) can sense nutrients, supported by the discovery of ‘taste’ receptors throughout the GIT [13,14,15,16,17].

**Figure 1:**
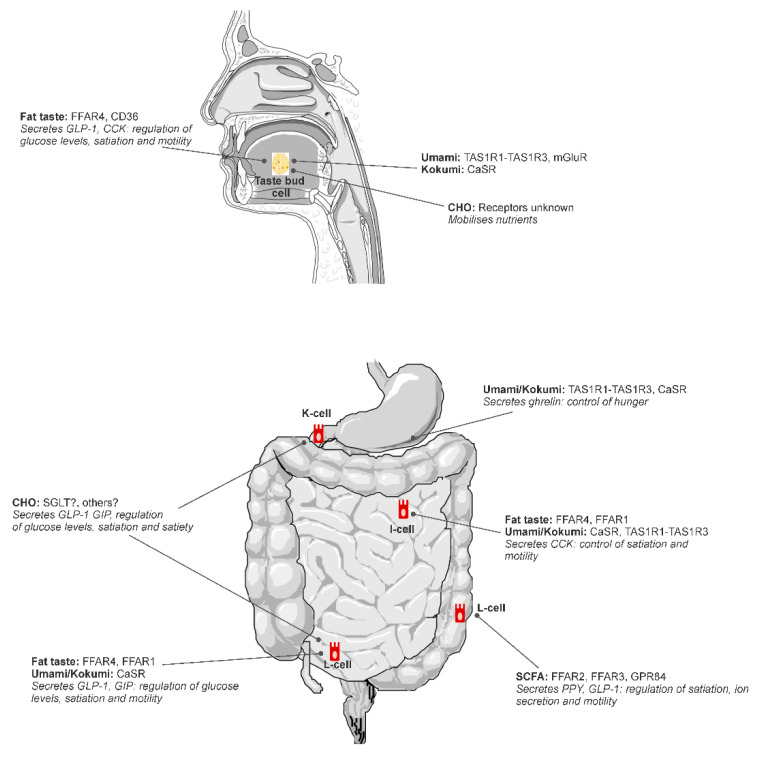
A diagram of putative and potential alimentary taste receptors throughout the alimentary canal. Reproduced from [18].

Putative tastes that have been proposed to fit into the alimentary taste classification include umami and fat (Figure 1), with further research required to confirm carbohydrate and kokumi (see [2]). Fat is the most scientifically mature non-traditional taste as illustrated by this review. Kokumi and carbohydrate taste are both emerging areas with more research required to achieve the level of sophistication of knowledge that exists for fat taste.

## 4. Macronutrient Fat: Fat Taste and Mouthfeel

Fat is one of the essential components of human diet and is necessary for the maintenance and function of many human processes. However, it is well established that overconsumption of fat has negative health implications and is associated with increased risk of obesity and metabolic disease [19]. Dietary fat consumption and energy homeostasis are regulated, in part, by fat sensing mechanisms during and following ingestion. Fat sensing is the ability to detect the presence of dietary fat in ingested foods in the mouth, throat, and GIT, which triggers a multitude of signals and processes to prepare the body for metabolism and satiety. 

Fat is a satiating nutrient where, in general, the more fat is consumed, the more satiated an individual will feel [20]. Fat intake is regulated by providing negative feedback to hunger signals or acting as a hunger ‘brake’, where the initial intake of fat slows subsequent intake until the individual reaches a point of satiation and a meal is ceased. It should be noted that the satiating power of fat does decrease when presented in mixed composition foods [21,22], although this does not undercut the importance of the role of fat in energy regulation. Therefore, it is important to understand the mechanisms involved in fat sensing in facilitating energy overconsumption and the pathogenesis of obesity. There are two main signaling mechanisms for fat sensing. First is the mouthfeel of TAG within the oral cavity where it imparts a textural quality [23]. The other is the detection of FA throughout the alimentary canal via FA sensing receptors [24,25]. It is possible that these sensory modalities complement each other to provide a full sensory perception of fat. It should be noted that fat also harbors odorous properties of foods, although these are usually fat-soluble compounds within the lipid matrix of a food and not necessarily the fats themselves. 

Most of the dietary fat that is consumed by humans is made up of TAG. Free fatty acid (FFA) may occur in quantities of less than 1% in most foods of the current food supply [26,27] due to modern refining processes and storage solutions. It is speculated that FFA may have been more abundant in foods in earlier periods of human history, so that the perception of naturally occurring FFA in food was an important gustatory function. Regardless, under normal circumstances, TAG is partially hydrolyzed into diglyceride (DG), monoglyceride (MG), and/or FFA by lingual lipase in the oral cavity, gastric lipase in the stomach, and a range of pancreatic lipases in the small intestine [28]. This demonstrates that even though the dietary fat in food is mostly comprised of TAG, it is still able to activate FA sensing pathways throughout all points of the alimentary canal. Thus, all dietary fat can trigger the satiety cascade upon ingestion, well prior to absorption of FA into the bloodstream via the small intestine. 

### 4.1. Triacylglycerol

TAG mouthfeel is the initial mode of fat sensing that occurs during an eating event as most dietary fat is comprised of TAG [26,27]. The associated textural properties of fat depend on the structure of the fats and the food matrix. These may be perceived as moistness, juiciness, smoothness, thickness, or crispiness depending on the role of fat in the food [29]. While the mechanisms for the sensing of each of these textural attributes may differ, they likely trigger the firing of oral responsive neurons. Unimodal neurons dedicated to the perception of viscosity in foods are present within the oral cavity [30], and more viscous foods are associated with greater feelings of satiety compared to non-viscous foods. For example, Marciani et al. showed that high-viscosity meals are more likely to slow gastric emptying and increase self-reported perception of satiety when nutrient loads are equal [31]. In addition, food with textures that require increased mastication–namely, crispiness and thickness for fatty food—increase the oro-sensory exposure time, thus allowing greater opportunity for sensory stimulation and signaling in the oral cavity [32,33]. However, the neurons involved in texture signaling are not necessarily specific to fat and may be influenced by other textural food components.

Non-hydrolyzed TAG does not aid in the regulation of food intake within the GIT. Matzinger et al. conducted a randomized crossover trial to assess the influence of TAG on appetite when infused directly into the GIT—bypassing the oral cavity and stomach—in 36 healthy male subjects [34]. Infusion of TAG into the duodenum reduced subsequent food intake compared to the control saline infusion. However, when TAG was infused with 120 mg of tetrahydrolipstatin, a lipase inhibitor, subsequent food intake was comparable to that of the control infusion. This highlights that TAG requires hydrolysis into FA via digestion before it can be sensed in the GIT.

### 4.2. Fatty Acid Sensing

FA sensing is the detection of FFA by FA receptors and the role of fat sensing in the alimentary canal is to signal the body in preparation for fat metabolism and energy homeostasis. This usually occurs subconsciously, particularly in the modern food environment where the proportion of FFA in dietary fat is relatively low compared to TAG [26,27], although recognition as a taste may occur in the oral cavity at slightly higher concentrations than would normally be found in food [25]. Very high concentrations may lead to epithelial irritation [35], although this is independent of FA taste sensing mechanisms.

Multiple receptors have been identified as candidate receptors for FA sensing in the alimentary canal including FA transporter CD36; G-protein coupled receptors (GPR) FFAR1, FFAR2, FFAR3, FFAR4, GPR84; and Delayed Rectifying K+ (DRK) channels [11,36,37,38]. Most of these receptors are present throughout the entire alimentary canal, although FFAR1 and FFAR3 have not been identified on taste bud cells in the oral cavity and are likely to only be present in the GIT in humans [39]. FA receptors are embedded on taste bud cells (TBC) in the oral cavity—specifically, within fungiform, foliate, and circumvallate papillae [39]—and enteroendocrine cells within the GIT [40].

Activation of FA receptors triggers a complex cascade of cellular events that result in hormone release and gut–brain signals via the vagus nerve, ultimately contributing to satiation and satiety [41]. Each receptor has similar, yet distinct, roles in the regulation of energy homeostasis [40], and there may also be some autocrine signaling between receptors within a cell [42]. FFAR4, previously known as GPR120, binds to medium-chain fatty acid (MCFA) and long-chain fatty acid (LCFA), with a greater affinity for LCFA [43]. The activation of FFAR4 by LCFA or FFAR4 agonists such as potent agonist GSK137657A in isolated mouse circumvallate papillae tissue triggers the release of glucagon-like-peptide (GLP-1) [44]. Similarly, FFAR4 expressed on enteroendocrine cells from mouse small intestine also triggers the release of GLP-1 and peptide tyrosine tyrosine (PYY) when activated [45], and FFAR4 knockout mice have reduced systemic release of GLP-1 following FA exposure [46]. CD36 is a FA translocator, where it transports LCFA through membranes to activate cellular signal cascades. It has a role in the release of oleoylethanolamide (OEA), which is a potent appetite regulator [47], via a cascade of intracellular signaling with peroxisome-proliferator-activated receptor (PPAR)-α [48]. There is also evidence in mice that CD36 may mediate the release of cholecystokinin (CCK), where LCFA infused into the stomach led to a greater release of CCK in wild-type mice compared to CD36 knockout mice [49], which may reflect coordinated crosstalk between CD36 and FFAR4 [42]. FFAR1, embedded within enteroendocrine cells, binds to MCFA and LCFA [43]. Following activation by LCFA, FFAR1 mediates the release of GLP-1 and GIP from L- and K-cells [50], and CCK in I-cells isolated from wildtype, but not FFAR1 knockout, mice [51]. FFAR2 and FFAR3 are involved in the chemoreception of short-chain fatty acid (SCFA) and GPR84 in the chemoreception of MCFA [11]. There is little evidence to support a role in satiety mediated by FA sensing from these receptors. Rather, their purpose seems to more related to the detection of FA that are produced from gut bacteria in regulating inflammation [52].

### 4.3. Individual Differences in Fatty Acid Sensing and Implications

There is large variation in FA sensing within and between individuals, with demonstrated differences in interindividual ability to detect FA ranging in concentrations up to as large as 1000-fold (0.02 mM to 20 mM) [22,53,54,55,56,57]. A test-retest analysis of fat taste sensitivity tests revealed high within day consistency (ICC = 0.80–0.88), whereas tests conducted at the same time across different days were only moderately consistent (ICC = 0.60–0.69), suggesting that fat taste sensitivity varies from day-to-day within an individual [58]. Variation in FA sensing is largely regulated by acute and habitual intake of dietary fat. Multiple dietary interventions have demonstrated that habitual low-fat intake increases fat taste sensitivity and, conversely, habitual high-fat intake attenuates sensitivity [59,60], even when body weight, gender, age, and genetics are controlled [61]. Following a recent analysis of the latter study, it was proposed that this may be due to the regulation of FA receptor gene expression, where it was found that an average reduction of approximately 20% energy from dietary fat for eight weeks resulted in the upregulation of *FFAR4* expression, the gene that encodes for FFAR4, by approximately 38% [62]. It has been hypothesized that these changes to sensitivity following dietary modification also occur in the GIT, although this has not been studied. Variation of FA sensitivity is an important phenomenon because it mediates how FA sensing influences appetite. Individuals with attenuated FA sensing have reduced signaling and a delayed satiety cascade [25], therefore they are intuitively more likely to consume excess energy and become overweight or obese.

FA sensing occurs in all regions of the alimentary canal and each autonomously trigger the satiety cascade. Research from our group demonstrated that FA sensing in the oral cavity, without exposure in the stomach or GIT, was able to influence self-reported perception of satiety [63]. A FA oral rinse increased the perception of fullness and reduced the perception of hunger compared to the control rinse. As for the GIT, French et al. conducted a study where multiple different FA were infused on separate occasions directly into the small intestine, thus bypassing the oral cavity and stomach [64]. All FA infusions increased self-reported perception of satiety, reduced subsequent meal intake, and triggered a greater release of serum CCK compared to the saline control, with linoleic acid (C18:2) demonstrating the strongest effect. These studies demonstrate the independent ability of tissues throughout the alimentary canal to sense FA and stimulate satiety. 

**Figure 2:**
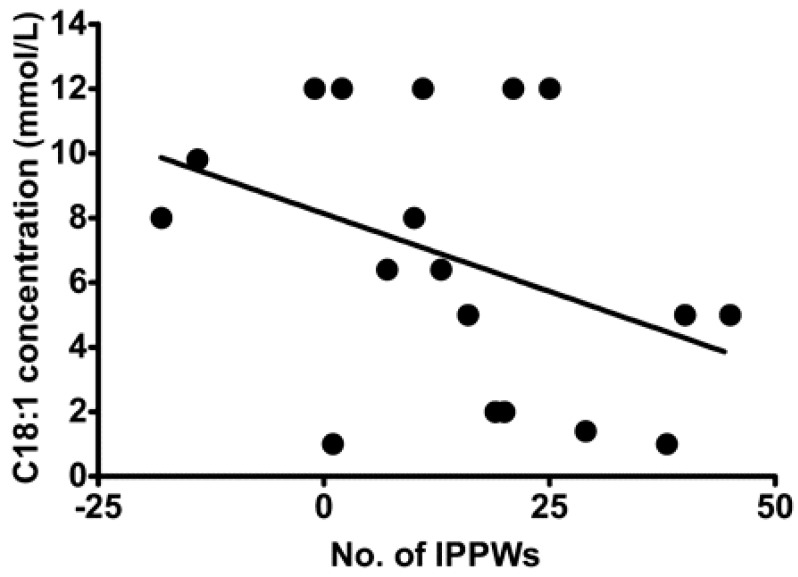
Relation between oral detection thresholds for oleic acid (18:1) and total number of isolated pyloric pressure waves (IPPWs) during 90-min intraduodenal infusions (0.78 kcal/min) of saline and oleic acid (18:1) in lean (*n* = 8) and overweight or obese (*n* = 11) subjects. Taken from [62].

Despite being able to act independently, the chemoreception of FA in the oral cavity and GIT are intrinsically linked [65] with various studies demonstrating an association between oral FA chemoreception and GIT response to FA [60,66,67]. Stewart et al. assessed isolated pyloric pressure waves (IPPWs) during an intraduodenal infusion of oleic acid (C18:1) over 90 min in eight lean and 11 obese males [68]. IPPWs slow gastric emptying and are stimulated by small intestinal exposure to FA, and thus a higher number of IPPWs suggest a gut that is more sensitive and responsive to FA. The study reported a relationship between the total number of IPPWs following C18:1 duodenal infusion and C18:1 taste threshold (Figure 2), suggesting that sensitivity in the oral cavity and GIT are associated. This is currently the strongest evidence to support the concept of the coordinated activity of FA sensing throughout the alimentary canal.

### 4.4. Fatty Acid Sensing, Satiety, and Diet

The ability of fat to stimulate satiation and satiety is likely to be an important regulator of energy intake and can vary widely depending on circumstance. For example, Keast et al. assessed subsequent intake of an *ad libitum* lunch after consumption of isoenergetic breakfasts of varying macronutrient composition [69]. In 24 participants, the high-protein breakfast caused the greatest level satiety. However, when stratified by fat taste sensitivity, individuals that were hypersensitive to fat taste (*n* = 14) consumed the least *ad libitum* lunch following the high-fat breakfast rather than the high-protein breakfast. This supports the concept that individuals have different satiety responses following food intake, which depend on their ability to sense nutrients in food. This may also change within an individual given that taste sensitivity varies day-to-day depending on recent meal intake [58,60].

Acute feeding studies have shown that intake of a high-fat meal/food leads to a greater release of satiety hormones in the gut and greater perceptions of satiety compared to lower fat meals/foods [66,70,71,72]. In one crossover trial, 16 overweight and obese participants (11 female) were provided with isoenergetic high-fat or high-carbohydrate breakfasts on separate days, then measured postprandial gut peptides and self-reported appetite over 180 min [71]. The high-fat meal led to a greater rise in GLP-1 and PYY compared to the high-carbohydrate meal, and self-reported ratings of satiety indicated that the high-carbohydrate meal was more satiating, although there was no significant difference between the two meals. Another study compared the effect of carbohydrate and protein meals with additional fat on plasma GIP in eight lean participants (four female, four male) [70]. Both the carbohydrate + fat meal and the protein + fat meal led to a greater rise in plasma GLP compared to the carbohydrate or protein meals without fat, respectively. However, as these meals were not isoenergetic, it does not indicate relative satiation of fat compared to other macronutrients. A study in eight obese females assessed the effect of plasma PYY—specifically a truncated version of PYY, PYY_3-36_—following intake of a high-fat, high-carbohydrate or high-protein meal over 180 min [72]. The high-fat meal caused the greatest increase in PYY, with at least 30% greater postprandial PYY levels over the other meals at 15–30 min. Another trial on 16 healthy men and 16 obese men was conducted where participants were fed a high-fat, high-carbohydrate, and high-protein meal on separate days, and gut hormone response and perception of satiety were measured over 180 min [66]. The high-fat meal caused greater perceived fullness and reduced perceived hunger compared to the high-carbohydrate meal and was similar to the high-protein meal. There was also a greater release of PYY following the high-fat meal compared to the other meals, but not for CCK. Finally, multiple studies have shown that acute fat intake has a similar or reduced ability to suppress ghrelin compared to other macronutrients [66,71,73,74]. Together, these studies highlight the effective capacity for fat to influence GLP-1, GIP and PYY release and self-reported perception of satiety, but is a relatively weak suppressor of ghrelin. 

Sham-feeding studies can be used to expose the oral cavity to fat stimuli while preventing the swallowing of food, thus testing the effect of fat sensing in the oral cavity without the influence of fat sensing in the gut. However, only a few studies have assessed the effect of sham-feeding fats on satiety or food intake. One study compared the effects of sham-feeding and consumption of a modest-fat meal in 10 healthy subjects (six female), and assessed CCK and pancreatic polypeptide (PP) over 90 min [75]. Compared to the control rinse (water), sham-feeding of the meal resulted in a greater release of CCK but not for PP. The consumption of the meal had a far greater effect on CCK and PP than sham-feeding. This shows that sham-feeding does influence some metabolic activity in the gut, but the effect appears to be small compared to actual ingestion. Another study that assessed the effect of sham-feeding of both a solid and liquid high-fat meal on 10 lean and 10 obese females yielded similar results, with a marked increase in plasma CCK and PP following sham-feeding of either meal [76]. However, this study only compared the effect to baseline levels of gut hormones, so there was no control meal for comparison. A randomized crossover trial by Costanzo et al. provided an oleic acid (C18:1) oral rinsing solution to 31 healthy participants and assessed their perception of satiety over 180 min [63]. Compared to the control rinse, the C18:1 oral rinse increased feelings of fullness and decreased feelings of hunger. This demonstrates that FA sensing in the oral cavity is still able to influence satiety, although it should be noted that this was only measured as self-reported satiety and not gut hormones.

There is substantial evidence to demonstrate the role of FA sensing in the oral cavity being associated with dietary habit [7]. Multiple studies have reported an association between fat taste sensitivity and dietary fat intake where, in general, hypersensitive individuals are less likely to consume excess energy—particularly from high-fat foods—compared to hyposensitive individuals [53,54,57,59,60,77,78,79,80].

There is also strong evidence to demonstrate the influence of FA sensing in the GIT on dietary intake [81]. A study by Feltrin et al. compared the effect of decanoic acid (C10:0) and lauric acid (C12:0) infusion into the duodenum on *ad libitum* buffet food intake after 90 min in 8 healthy male participants [82]. Energy intake was substantially reduced after the C12:0 infusion compared to the C10:0 and saline control infusions, suggesting that chain lengths of 12 (and above) are necessary for FA sensing in the GIT to trigger the satiety cascade. Another study by Feltrin et al. administered C12:0 and C18:1 infusions into the duodenum of 13 healthy men and measured *ad libitum* buffet food intake after 60 min [83]. Energy intake from the buffet meal was least following the C12:0 infusion compared to the C18:1 and saline control infusions. Interestingly, C18:1 infusion did not reduce food intake compared to the control, even though longer chain length FA are reported to have the greatest satiating effect [34]. A similar study had opposing findings, with C18:1 infusion in the duodenum reducing buffet meal intake after 90 min compared to a saline control infusion in 8 lean males [68]. The difference in meal intake between conditions was less in the 11 overweight or obese male subjects, suggesting that body mass or FA sensing sensitivity might modify the strength of this effect.

Despite strong evidence to suggest that fat sensing regulates satiety and dietary intake, the evidence for the link between attenuated fat sensing and obesity remains contentious. Various human studies that have assessed the link between fat taste sensitivity and body weight have reported that participants who were more sensitive to FA were more likely to have lower BMI than less sensitive individuals [53,55,78,79,80,84]. On the other hand, some studies have failed to find such associations [54,56,60]. A meta-analysis of 7 studies, conducted to assess the relationship between fat taste sensitivity and body weight [85], reported a minimal difference in fat taste threshold between lean vs. overweight and obese individuals (standard mean difference 0.19 [95% CI: −0.09, 0.47]) suggesting that fat taste sensitivity is not mediated by body mass. However, this analysis was based on a limited number of studies and did not include a wider range of methodologies and conclusions. A number of new studies have been published since the original meta-analysis, so an updated meta-analysis is warranted. 

Similarly, the literature suggests that body mass is not associated with attenuated sensitivity throughout the whole alimentary canal. One study compared the effect of a high-fat meal on plasma GLP-1 and GIP between 6 lean and 6 obese women matched for age [86]. They reported no difference in plasma GIP and minimal difference in plasma GLP-1 between lean and obese subjects following high fat meal intake, suggesting that obesity has minimal influence on the effect of fat-mediated release of satiety hormone within the GIT. Another study compared the effect of a high-fat pasta meal matched to 30% of each subject’s estimated daily energy requirement between 16 lean and 16 obese men [66]. There were trends for a greater initial reduction in hunger (*p* = 0.08) and increase in fullness (*p* = 0.09) in lean individuals compared to obese individuals following intake of the high-fat meal. Additionally, there were no differences in the AUC for either marker of perceived satiety between the obese and lean subjects. Similarly, no differences were reported in CCK, PYY, or ghrelin between the obese and lean subjects following the high-fat meal. French et al. reported similar findings from a preliminary study. Eight obese and 7 age and sex-matched healthy weight participants were given a high-fat soup (containing 30 g of margarine) and gastric emptying, mouth to caecum transit time (MCTT), plasma CCK, and perception of satiety were assessed [87]. They observed no difference in gastric emptying or MCTT between healthy-weight and obese subjects. Obese subjects did have higher CCK levels than healthy-weight subjects and a reduced feeling of hunger following the high-fat soup ingestion. Together, these studies suggest that the GIT response to dietary fat does not differ substantially between lean and obese individuals. However, it should be noted that these studies did not take sensitivity to satiety hormones into consideration. It is possible that although the hormonal response is comparable in lean and obese individuals, their responsiveness to these hormones may differ.

As discussed above, there is extensive research on the sensing and implications of dietary fat consumption, from pre-ingestion FA sensing in the oral cavity by taste receptors, to post-ingestive signaling by ‘taste’ receptors throughout the GIT and subsequent hormonal modification and satiety. Fat taste has a prominent role in the regulation of dietary fat intake and future studies should focus on the use of fat stimuli as a potential mechanism for appetite regulation.

## 5. Macronutrient Protein: Umami and Kokumi Tastes

Proteins are highly diverse in composition, found in all organ systems of animals and plants, and are involved in key functions enabling life. Protein is composed of 20+ amino acids, nine of which are considered essential to humans, and must be obtained via diet. It was a requirement for continued survival that if a species came upon a food source that contained protein, there were sensing mechanisms to identify the protein and encourage consumption of the food. Foods that contain proteins also naturally contain peptides and amino acids, and proteases in saliva start to hydrolyze protein to release peptides and amino acids. It is the peptides and amino acids that allow protein to be sensed and in humans, the two perceptual qualities associated with protein are umami and kokumi, which may enable the regulation of protein intake [88,89,90].

There has been considerable research on umami taste from the early 2000s, aided by the identification of a glutamate taste receptor [10]. Umami, which is described as savory and delicious, is predominately stimulated by the ionic form of the amino acid glutamic acid, L-glutamate, or more precisely the sodium salt form of L-glutamate. The umami quality is synergistically enhanced by ribonucleotides, inosinate, and guanylate monophosphate (IMP and GMP) [91]. Taste receptors responsible for detecting L-glutamate include the T1R1/T1R3, and metabotropic glutamate receptors (mGluRs) [10,92,93].

Kokumi is a relatively new taste concept following the recognition of γ-glutamyl peptides found in foods including legumes, some cheeses, and fermented foods [94]. In isolation, koku stimuli elicit minimal taste, but instead enhance thickness, mouthfeel, and continuity when mixed with other taste stimuli such as umami, sweet, and salty stimuli [12,95]. Koku compounds (γ-glutamyl peptides) have been shown to activate a calcium-sensing receptor (CaSR) in taste cells on the tongue [12,96,97], however, the mechanisms by which kokumi enhances other basic tastes on a molecular level has not been elucidated.

These proteins signaling mechanisms are first initiated in the oral cavity via the T1R1/T1R3 L-glutamate taste receptor, and the CaSR activation via y-glutamyl peptides; importantly, the same umami taste receptors and the CaSR are found throughout the gastrointestinal tract (GIT), with the post-ingestive activation triggering the release of hormones that play a role in modulating satiation and hunger [98,99,100].

### 5.1. Sensing Glutamate and γ-Glutamyl Peptides Throughout the Alimentary Canal

Studies in rats suggest that there is an existing sensing system for glutamate in the gastric mucosa, so that when monosodium glutamate (MSG) is infused into the stomach, there is an increase in the firing of the vagus afferent nerves, which interestingly was not seen in response to the 19 other amino acids, nor sodium chloride [17]. From this, it was hypothesized that the surface of the stomach can sense glutamate [17]. The physiological response to this GIT glutamate sensing includes several hormones that play a role in moderating food intake. The predominant hormones include ghrelin and cholecystokinin (CKK), which are likely to contribute to slowing gastric emptying, moderating motility of the intestine, and stimulating secretions in other organs (i.e., pancreatic and gallbladder secretions) [101,102].

In vitro animal studies have shown that umami and amino acid taste receptors (T1R1-T1R3) detect umami stimuli (MSG) on a gastric ghrelinoma cell line and play a role in the release of ghrelin [101]. T1R1-T1R3 and CaSR receptors are similarly expressed on cells that release CCK, and are involved in CCK release, thus having a satiating effect [14,103]. The T1R1-T1R3 is expressed in intestinal endocrine cells and is activated by a broad range of L-amino acids, which promotes CCK secretion. This activation and CCK secretion is further enhanced with the addition of IMP [14]. Interestingly, enhancement of CCK secretion in the presence of IMP is similar to the synergistic effect on taste perception seen when IMP is applied with L-glutamate in the oral cavity; it may be that a synergistic effect also occurs with CCK secretion in the GIT [14]. Moreover, recent research has shown that kokumi active γ-glu peptides have an in vitro dose response release effect on CCK and GLP-1, providing further evidence that CaSR activation by kokumi active peptides is likely involved in the release of hormones responsible for appetite and food intake regulation [103]. Altogether, these animal studies suggest the umami and koku stimuli play a role in the release of digestive hormones.

It is well known that protein mediates the release of hunger and satiety-related hormones [98], thereby contributing to the regulation of food intake. The effect is enhanced with the addition of umami tasting stimuli (MSG or MSG + IMP) [98,100], although the impact on *ad libitum* food intake and subjective appetite ratings is mixed [98,100,104,105,106,107]. A recent study found that the addition of MSG to a soup alone did not affect food intake or blood hormones, however, when consumed in combination with protein, changes in subjective ratings (increased fullness, reduced desire to eat, and reduced appetite) were seen in conjunction with a decrease in blood glucose and increase in plasma insulin and C-peptide [98]. Moreover, the addition of MSG to a carbohydrate-based pre-load soup did not alter post prandial blood glucose levels or appetite ratings in healthy individuals, however, partial energy compensation at the subsequent *ad libitum* lunch was observed between the low energy dense savory pre-load soup in comparison to the sweet pre-load, although the same effect was not seen in the high energy dense pre-load [99]. This suggests that it is energy density, rather than taste perception that may regulate food intake and post prandial glucose release [99]. However, the use of maltodextrin in both sweet and savory soups adds an aspect of carbohydrate content and carbohydrate taste stimulation to both conditions, potentially impacting the outcomes, as sensitivity to maltodextrin (stimuli for carbohydrate taste) has been shown to impact *ad libitum* consumption of complex carbohydrates [108], and is associated with habitual energy intake [109]. In contrast, Hosaka et al. found that following an MSG-containing liquid meal reduced postprandial glucose concentration and increased GLP-1 secretion in comparison to a NaCl control, indicating that MSG may influence satiety by stimulating the release of GIT hormones [100]. 

Accordingly, the evidence suggests that umami and kokumi receptors exist in the GIT, and the presence of stimuli in the GIT can lead to the release of digestive hormones that play a role in moderating the digestion process, satiety, appetite, and food intake.

### 5.2. Behavioral and Health Outcomes of Umami/Kokumi Stimuli

Umami and kokumi stimuli have been shown to have beneficial effects on appetite and satiety, particularly when combined with protein, through the potential modulation of digestion and satiety related hormone release post-ingestion, and thus food intake regulation [98,99,100]. Additionally, umami/kokumi taste perception and hedonic responses may be associated with protein intake regulation [98], because many high protein foods having an umami/savory flavor [110]. 

Savory (or umami) food liking, and preference is closely tied with the protein content of food, and high protein foods often (but not always) have umami characteristics [110]. The liking and preference of protein foods is also associated with the protein or nutritional status of the individual, however, links with umami and kokumi taste perception require further research [111,112]. Studies have shown that when participants are in a protein deficit or have an overall poor nutritional status, there is a preference for increased concentrations of MSG [111], and higher intake of savory protein foods [112], thereby regulating their protein intake. Unfortunately, the umami or kokumi taste sensitivity of subjects in these studies was not assessed, so the role of taste perception in protein intake regulation is unclear. 

Prolonged consumption of MSG-containing soup decreases umami taste perception, desire for savory foods, and intake of savory foods in healthy populations [107], potentially due to effects on appetite occurring post-ingestion. Interestingly, obese adolescents [113] and obese adult women have a lower sensitivity to MSG and prefer higher concentrations of MSG when compared to healthy weight women [114]. Obese populations have been found to consume a higher proportion of daily energy from salt-, fat-, and umami-dominant foods than healthy weight individuals [115], potentially contributing to their reduced umami sensitivity. This reduced MSG sensitivity is not observed in healthy weight populations, in fact, the inverse has been reported, with healthy participants who are more sensitive to MSG at threshold concentrations having a greater liking and preference for high protein foods in comparison to less sensitive participants [116]. Although further research is required, it appears that obesity is associated with a reduced umami taste perception and preference toward higher concentrations of MSG and umami/savory flavored food. In some cases, this could be attributed to the nutritional status of overweight/obese participants, as poor nutritional status and protein deficits have been associated with the preference of higher concentrations of umami stimuli [111,112]. It may also be that higher concentrations of umami stimuli are required for both taste detection/perception to occur, and for umami GIT receptor activation in order to elicit the same physiological response seen in healthy weight individuals. 

**Figure 3:**
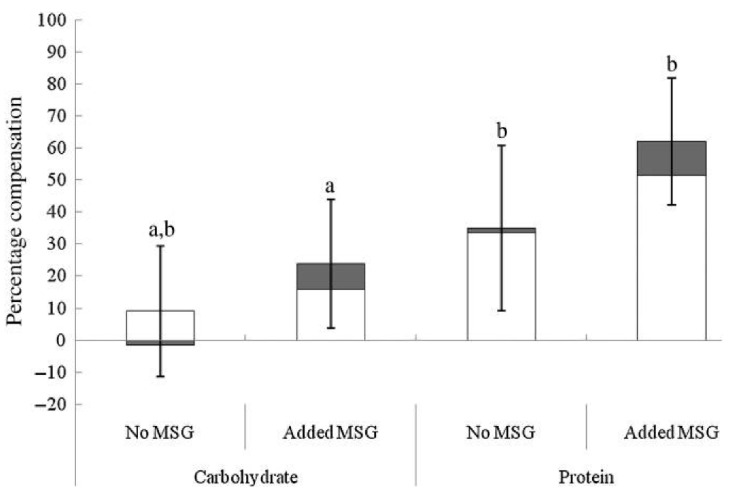
Energy compensation at an *ad libitum* test meal (pasta main course (□) and ice cream dessert (■)) after fixed consumption of high-energy carbohydrates and high-energy protein soup pre-loads with and without added monosodium glutamate (MSG). Values are means, with standard errors represented by vertical bars. ^a, b^ Mean values with unlike letters were significantly different (*p* ≤ 0.05; within-subjects Bonferroni-corrected contrasts). Reproduced from [105].

Although taste perception and umami/savory food preferences in obese and overweight individuals appear to differ from healthy weight counterparts, there are also studies supporting the concept of using umami stimuli to decrease food intake and enhance satiety. In overweight and obese women, the addition of MSG to a soup pre-load reduced total energy intake and energy intake from high-fat savory foods at a subsequent *ad libitum* lunch and tended to lower energy intake at a further afternoon snack, in comparison to the no MSG control [117]. In support of this, when energy content of a pre-load soup is increased through the addition of protein, participants can adjust their energy consumption at an *ad libitum* meal more precisely than when energy is increased using carbohydrates; this energy compensation effect is enhanced further with the addition of MSG to the protein pre-load, Figure 3 [105]. Interestingly, although energy compensation occurred, this was not associated with an enhancement in satiety or reductions in appetite ratings prior to the *ad libitum* meal [105]. This is supported by a study showing that prolonged MSG consumption leads to a reduction in both desire for, and intake of savory foods without altering subjective appetite and hunger ratings during the *ad libitum* meal [107]. Possibly, this adjustment of energy intake during the *ad libitum* meals reflected post-ingestive appetite regulation, potentially through activation of GIT glutamate receptors promoting the release of digestive hormones, rather than impacting subjective satiety and appetite, as supported in previous studies [14,100,103]. In contrast, the presence of umami flavored food in the oral cavity results in an immediate increase in appetite, followed by an increase in post-ingestive satiety, potentially indicating a biphasic effect of MSG [104], however, this enhancement in satiety has not consistently resulted in subsequent decreased energy intake [106].

An alternative health promoting role of umami/kokumi stimuli is to promote health in the elderly, where there are some promising results. A common problem that can favor under nourishment in the elderly is a decline in taste perception, which ultimately contributes to a reduction in appetite and food intake, leading to loss in body weight, predisposition to comorbidities, and reduced quality of life [118]. It is similarly common for the elderly to experience a dry mouth caused by a reduction in salivary output that can contribute to these taste sensation disorders [119]. It has been shown that salivary stimulation by umami stimuli can relieve dry mouth symptoms and improve oral functioning including taste sensation [120], increasing the mucosa to prevent bacterial contamination in the oral cavity [121], and consumption of umami flavored food may enhance overall dietary intake and nutritional status in the elderly [122]. 

Thus, the use of umami stimuli to alleviate disorders in oral functioning that contribute to malnutrition in the elderly may represent another potential health benefit of umami or kokumi tastants in a nutritionally at-risk population group. Moreover, physiological, and behavioral evidence suggests that the addition of, or the flavor of, MSG and kokumi in food can regulate protein intake, enhance satiety, and potentially moderate food intake through the modulation of digestive hormones. However, further research is required, particularly for use in overweight and obese populations, and other sub-population groups such as those with type 2 diabetes, which would help develop our understanding of the potential clinical application of umami and kokumi stimuli. To further develop our understanding of the clinical applications of MSG and kokumi on long-term food intake, appetite, and satiety regulation (including modulation of digestive hormones), long-term clinical trials assessing these associations are required. Additionally, studies researching associations between umami and kokumi taste perception and these behavioral and physiological outcomes are needed. Finally, as there is little research investigating the role of oral stimulation by umami and kokumi compounds on cephalic phase responses, further investigation is required. 

## 6. Macronutrient Carbohydrate: Sweet and Carbohydrate Taste

There are three main classes of carbohydrate: mono/disaccharides (sugars), oligosaccharides, and polysaccharides, with the chain length of the compound the determining factor for class membership. To add to the complexity, some carbohydrates provide energy, while others cannot be metabolized and are classified as dietary fiber. Some carbohydrates are soluble in aqueous solutions and others remain insoluble. For the purpose of this review (unless otherwise stated), carbohydrate taste stimuli are soluble oligosaccharides, usually maltodextrin. Maltodextrin is a complex carbohydrate that has a variable starch-based structure composed of d-glucose chains linked by glycosidic α-(1–4) and α-(1–6) bonds [123].

As opposed to simple carbohydrates (sugars elicit sweet taste, for review see Trumbo et al. [124]), complex carbohydrate taste research is in its infancy. Indeed, it has long been assumed that maltodextrin is invisible to taste [125,126], and as such has been used as tasteless caloric ingredients in flavor-nutrient conditioning studies [127,128,129]. However, there is evidence demonstrating that rodents (e.g., rats, mice, gerbils, hamsters) and even some non-human primates are attracted to the taste of maltodextrin [130,131]. Sclafani and Mann [132] found that the preference profiles for five different carbohydrates varied as a function of concentration in three-minute two-bottle choice tests. At low molar concentrations, rats preferred maltodextrin to sugars (maltose, sucrose, glucose, fructose), whereas at higher molar concentrations, rats preferred sucrose and maltose in comparison to maltodextrin [66]. 

Recent physiological evidence from exercise science suggests that performance is improved after participants rinsed their mouth with solutions containing maltodextrin compared to non-nutritive sweetener (NNS) control solutions [133]. Additionally, Chambers et al. [134] investigated the cortical response to oral maltodextrin and glucose solutions, revealing a similar pattern of brain activation in response to both solutions including brain areas involved in the reward system (i.e., activates brain reward centers in orbitofrontal cortex and striatum similar to oral glucose, which were unresponsive to NNS). Psychophysical research also provides evidence that humans perceive maltodextrins and that sensitivity to simple carbohydrates is independent of that to complex carbohydrates [109,135,136,137,138]. Together, these findings provide evidence of taste transduction pathways that respond to maltodextrin independently to those for sweet taste [139]. What follows is an overview of relevant human psychophysical studies using maltodextrin.

### 6.1. Carbohydrate Taste Psychophysics

Research conducted at Oregon State explored individual differences in the taste perception of carbohydrates, individual differences in activity of salivary alpha-amylase, and the role that salivary α-amylase plays in the taste perception of glucose polymers [135,136]. To assess individual differences in the taste perception of carbohydrates, participants tested six stimuli (three glucose polymers [10% maltodextrin preparations with varying levels of dextrose equivalence (DE of 5, 10, and 20)] and three prototypical stimuli [10% glucose monohydrate, 6% sucrose, and 0.6% sodium chloride]). Maltodextrins were shown to be associated with lower average intensity ratings compared with the sweet and salty stimuli, and the intensity ratings of the maltodextrins highly correlated with one another. Furthermore, the taste responsiveness to the maltodextrins showed greater variability across individuals when compared to the sweet and salty stimuli. 

The same research group subsequently investigated taste detection and discrimination of maltodextrins with varying chain length while inhibiting α-amylase activity and additionally explored the effects of a sweet taste inhibitor (lactisole) on taste discrimination. To investigate the taste discrimination of maltodextrins, 22 participants were presented with 6% and 8% samples of two maltodextrins (DP 7 and DP 14) and maltodextrin polymer (DP 44), all spiked with 5 mM of acarbose (an α-amylase activity inhibitor). To investigate the effects of a sweet taste inhibitor (lactisole) on taste discrimination, participants were instructed to taste five samples (75 mM glucose, 75 mM maltose, 0.025 mM sucralose and two maltodextrins [DP 7 and DP 14]). The potentially confounding factor of salivary α-amylase activity was also inhibited by adding 5 mM acarbose to all samples. Controlling for α-amylase is important as the hydrolysis by-products of maltodextrin is glucose, which may activate sweet taste receptors [135,136]. It was found that participants could differentiate between the two maltodextrin oligomer samples, but not the maltodextrin polymer sample [136]. Furthermore, when lactisole was present in the samples, the detectability of the maltodextrin oligomers was not compromised, in contrast to the other samples. This supports the concept that oligomers such as maltodextrin have a taste transduction mechanism independent of the hT1R2/hT1R3 sweet receptor. 

Our research group used taste assessment methodology to assess if oral sensitivity to maltodextrin and oligofructose is independent of basic tastes [138]. This taste assessment methodology recruited 34 healthy adult participants to receive 12 samples (two oligosaccharides [maltodextrin, oligofructose], six sweeteners [caloric and NNS], and prototypical stimuli [sour, salty, umami and bitter]) over 28 sessions. Detection and recognition thresholds and intensity ratings were assessed for all stimuli. The outcomes showed that that oligosaccharides can be sensed in the oral cavity and that maltodextrin and oligofructose were highly correlated (r = 0.94–0.95), indicating that the oligomers access the same peripheral receptor mechanism [138]. It was also interesting that at lower concentrations of maltodextrin and oligofructose, there were no associations with sweet taste, but at higher concentrations, there was some overlap with sweet taste. It is unlikely that lingual amylase activity was responsible for increasing free sugars, thereby causing the association between sweet and carbohydrate taste as oligofructose is a fiber and not broken down by oral amylase. Accordingly, there appears to be an independent taste transduction pathway for oligomers at low concentrations, but sweet taste and carbohydrate taste may share some peripheral physiology at higher concentrations [138].

### 6.2. Behavioral and Health Outcomes of Carbohydrate Taste

**Figure 4:**
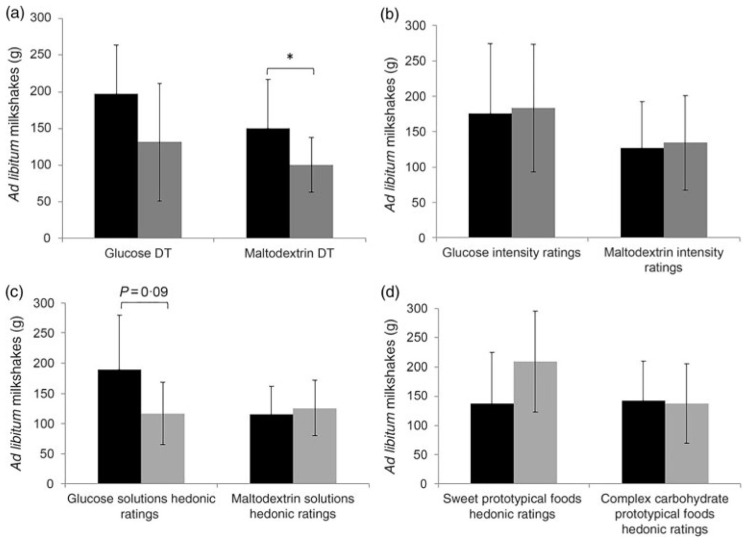
(**a**,**b**) *Ad libitum* milkshake intake means and standard deviations between more sensitive and less sensitive participants or those who experienced high and low intensity ratings. (**c**,**d**) *Ad libitum* milkshake intake means and standard deviations between participants with high hedonic ratings and low hedonic ratings for both sweet and complex carbohydrate solutions and prototypical foods. For sweet taste function and sweet hedonic ratings, comparisons were only made for sweet (glucose) milkshakes, and vice versa for complex carbohydrate (maltodextrin). * *p* = 0.01. DT = Detection threshold (reproduced with permission from the authors [108]).

Low et al. examined associations between carbohydrate (maltodextrin) taste sensitivity and *ad libitum* consumption of complex carbohydrate foods [108]. In this study, 51 adult females consumed two different iso-caloric pre-load milkshakes followed by an *ad libitum* intake of milkshakes (a sweet glucose-based milkshake and a non-sweet maltodextrin-based milkshake) in a crossover design. Detection threshold and suprathreshold intensity perception ratings for glucose and maltodextrin were collected as well as hedonic (rating of liking) ratings for glucose and maltodextrin and hedonic ratings for various sweet and complex carbohydrate foods. It was found that participants who were more taste sensitive toward maltodextrin consumed more maltodextrin-based milkshake compared to less taste sensitive participants, and this was independent of liking (Figure 4). Although there were variances in intake of maltodextrin-based milkshake, there were no significant differences in appetite ratings (i.e., decrease in hunger and prospective consumption, increase in fullness) between those who were more sensitive and less sensitive to maltodextrin. Maltodextrin sensitivity may be associated with increased consumption of carbohydrate foods although the mechanism remains unknown. The authors speculate that sensing carbohydrates (maltodextrin) may promote unconscious consumption due to the activation of specific brain regions involved with taste and reward [108]. 

Further research into the associations of oral complex sensitivity was conducted by Low et al. [109]. Using taste assessment methodology (detection threshold and suprathreshold intensity perception), participants tested two samples (maltodextrin and oligofructose). To determine sensitivity to the carbohydrate compounds, 34 participants (18 female) were grouped into tertiles: tertile 1 (participants experiencing higher sensitivity), tertile 2 (normal sensitivity), and tertile 3 (less sensitivity). This was done through assessing differences between the continuous variables (waist circumference, BMI, and habitual energy intake via quantitative FFQ) and the detection threshold ratings. The outcomes showed an association between carbohydrate taste sensitivity and consumption of complex carbohydrates. Experiencing strong taste intensity or being more sensitive to maltodextrin were associated with a greater energy and starch intake and also a greater waist circumference. The authors suggest that individuals with heightened oral sensitivity responses to maltodextrin may have developed preferences for complex carbohydrate flavors because of post-digestive nutritive cues (conditioned preferences), leading to a greater intake of energy and starch and consequently a larger waist circumference [109]. The body of research from Low et al. established the importance of assessing individual sensitivity to carbohydrates as it may influence other factors including taste intensity, waist circumference, energy intake, and consumption [108,109,138,140].

Carbohydrate taste research is in its infancy, with both animal and human data strongly indicating there are oral peripheral mechanism/s that respond to maltodextrins, that are associated with consumption, and the potential development of overweight and obesity. Future research should look at individual differences in ‘taste’ sensitivity to maltodextrins and incorporate advanced molecular biology techniques to identify the peripheral mechanisms along with downstream processing. If carbohydrate receptors are an accelerator of consumption when activated, understanding the structure of ligands may provide opportunities for new foods to help populations suffering from wasting, for example, cancer cachexia.

## 7. Conclusions

Taste receptors originally identified in the oral cavity have subsequently been located through the GIT, indicating that at least at the epithelium of the alimentary canal, there is commonality in peripheral sensing mechanisms. Taking this further, the concept of a coordinated macronutrient sensing throughout the alimentary canal seems logical. This involves multiple perceptual phenomena for each macronutrient, working in combination to tailor liking and preference of foods and regulate consumption through the activation of the satiety cascade. The macronutrient taste qualities working in combination may represent an important focus for future investigations on the links between the taste system and diet, given the variable outcomes of past studies, or qualities like sweet where no associations with diet have often been reported [7]. 

The extent and sophistication of research for fat, protein, and carbohydrate sensing is variable, with umami and fat tastes being mature fields, while kokumi and carbohydrate are in their relative infancy. A major area of interest is the directional difference in how the sensitivity of alimentary tastes moderate satiety and intake. Those more sensitive to the fat taste consume less fat and have lower BMI, while those who are more sensitive to maltodextrin (carbohydrate) appear to consume more carbohydrate and energy and have increased waist circumference. This indicates that individuals who are less sensitive to fat and more sensitive to carbohydrate may have more difficulty in achieving fullness without overconsuming fat, carbohydrate, and energy. More comprehensive studies are required including alimentary taste directed acute and habitual dietary interventions, satiety protocols, combined with molecular biology to understand the link between alimentary taste sensitivity, satiety hormones, diet, overweight/obesity, and taste receptor expression.
